# Digital three-dimensional visualization of intrabony periodontal defects for regenerative surgical treatment planning

**DOI:** 10.1186/s12903-020-01342-w

**Published:** 2020-12-01

**Authors:** Daniel Palkovics, Francesco Guido Mangano, Katalin Nagy, Peter Windisch

**Affiliations:** 1 Department of Periodontology, Semmelweis University, 1088 Szentkirályi Str. 47. 4th Floor, Budapest, Hungary; 2 Department of Prevention and Communal Dentistry, Sechenov First State Medical University, Bol’shaya Pirogovskaya Ulitsa, 19c1, Moscow, Russia; 3 Department of Oral Surgery, University of Szeged, Tisza L. Str. 64, Szeged, Hungary

**Keywords:** Periodontal diagnostics, Regenerative periodontal surgery, Three-dimensional treatment planning, CBCT, Segmentation, 3D radiographic image reconstruction

## Abstract

**Background:**

In the regenerative treatment of intrabony periodontal defects, surgical strategies are primarily determined by defect morphologies. In certain cases, however, direct clinical measurements and intraoral radiographs do not provide sufficient information on defect morphologies. Therefore, the application of cone-beam computed tomography (CBCT) has been proposed in specific cases. 3D virtual models reconstructed with automatic thresholding algorithms have already been used for diagnostic purposes. The aim of this study was to utilize 3D virtual models, generated with a semi-automatic segmentation method, for the treatment planning of minimally invasive periodontal surgeries and to evaluate the accuracy of the virtual models, by comparing digital measurements to direct intrasurgical measurements.

**Methods:**

Four patients with a total of six intrabony periodontal defects were enrolled in the present study. Two months following initial periodontal treatment, a CBCT scan was taken. The novel semi-automatic segmentation method was performed in an open-source medical image processing software (3D Slicer) to acquire virtual 3D models of alveolar and dental structures. Intrasurgical and digital measurements were taken, and results were compared to validate the accuracy of the digital models. Defect characteristics were determined prior to surgery with conventional diagnostic methods and 3D virtual models. Diagnostic assessments were compared to the actual defect morphology during surgery.

**Results:**

Differences between intrasurgical and digital measurements in depth and width of intrabony components of periodontal defects averaged 0.31 ± 0.21 mm and 0.41 ± 0.44 mm, respectively. In five out of six cases, defect characteristics could not be assessed precisely with direct clinical measurements and intraoral radiographs. 3D models generated with the presented semi-automatic segmentation method depicted the defect characteristics correctly in all six cases.

**Conclusion:**

It can be concluded that 3D virtual models acquired with the described semi-automatic segmentation method provide accurate information on intrabony periodontal defect morphologies, thus influencing the treatment strategy. Within the limitations of this study, models were found to be accurate; however, further investigation with a standardized validation process on a large number of participants has to be conducted.

## Background

Regenerative treatment of periodontal defects was first published in the early 1980’s [1]. Since the first concepts were formed on regenerative periodontal therapy, minimally invasive surgical approaches [2–4] and new biomaterials [5] have been introduced to enhance regenerative potential and reduce patient morbidity. A decision tree has been published by Cortellini to aid the decision-making process in the regenerative surgical treatment of intrabony periodontal defects. Among different site related factors, the morphology of the intrabony defect is the primary determining factor in the selection of the surgical technique and regenerative strategy [6].

Direct clinical measurements (probing pocket depth: PPD, gingival recession: GR, clinical attachment loss: CAL) [7] and intraoral radiographs: IRs) acquired with parallel long-cone technique [8, 9] are the main tools in periodontal diagnostics. However, there are limitations to the aforementioned methods. Clinical studies have demonstrated that clinicians constantly underestimated the extent of intrabony defects during direct clinical measurements. IRs provide a two-dimensional (2D) image, in which overlapping anatomical structures make it difficult to accurately determine the true three-dimensional (3D) defect morphology [10, 11].

The application of cone-beam computed tomography (CBCT) in periodontal diagnostics has been proposed by many authors [12–14]. A series of in vitro and in vivo studies have demonstrated that CBCT is superior to IRs in the detection of certain periodontal defects (i.e., furcation defects, three wall intrabony defects, midbuccal intrabony defects, or dehiscence-type defects) [15–22]; however, it is difficult to justify the cost–benefit ratio of the higher irradiation dose [23–25]. Therefore, CBCT should only be used for periodontal diagnosis if conventional radiographic methods do not provide a sufficient amount of information [26].

In the aforementioned articles, investigators examined periodontal defects on 2D images of the CBCT dataset [12–22]. Even though 2D images provide views in multiple orientations (sagittal, coronal, axial), the true 3D defect morphology is not visible. Therefore, articles have proposed the application of 3D virtual models in periodontal diagnostics and treatment planning [27–29]. Here, authors utilized basic thresholding algorithms for radiographic image reconstruction. Detailed, realistic 3D models could not be acquired, due to artefacts and scattering compromising image quality and the 3D renderings [30].

Various surgical fields in general medicine such as cardiac surgery [31] and orthopedic surgery [32] have utilized different semi-automatic image segmentation techniques to generate patient-specific digital three-dimensional renderings for diagnostic purposes. Different anatomical structures were separated into multiple regions of interest (ROI) for better understanding of the clinical scenario [31, 32]. Similar anatomy-based segmentation techniques have not yet been described for dental application.

The aim of this study was to present a novel semi-automatic segmentation method to acquire 3D virtual models of alveolar and dental structures for the treatment planning of minimally invasive periodontal surgeries and to evaluate the accuracy of the virtual models, by comparing digital measurements to direct intrasurgical measurements.

## Methods

### Patient selection

Four patients with six intrabony periodontal defects were enrolled in this preliminary study. The selected patients were diagnosed with Stage III/ Grade B periodontitis [33] and were in need of complex perio-prosthetic rehabilitation. The study was conducted with full accordance to the Declaration of Helsinki (2008) and was approved by the local ethical committee (Semmelweis University Regional and Institutional Committee of Science and Research Ethics, ref. no. 195/2017). Surgical interventions were performed with the understanding and written consent of each participant.

Inclusion criteria were: (1) *absence of general medical conditions*: previous irradiation therapy in the maxillo-facial area, uncontrolled diabetes, systemic steroid treatment, systemic bisphosphonate treatment, pregnancy; (2) *smoking status:* only non-smoking patients were included; (3) *oral hygiene:* full mouth plaque score (FMPS) ≤ 25%; (4) *residual inflammation*: full mouth bleeding score (FMBS) ≤ 25%. Mean age was 48.75 ± 14.82 years, 2 patients were male, and 2 patients were female. Three single rooted teeth (upper central incisor, lower second incisor, lower first premolar) and three multi rooted teeth (lower first molar, upper first molar, upper second molar) were enrolled into the present study.

### Image acquisition

Two months following initial periodontal treatment, CBCT scans were taken with I-CAT FLX^®^ (KaVo Dental GmbH, Bieberach an der Riß, Germany) 300 µm voxel size; 120 kV anode voltage; 36 mA x-ray tube current; 16 cm × 8 cm field-of-view. In all cases, prosthetic rehabilitation was planned with implant retained fixed partial dentures. Therefore, a large field-of-view (FOV) CBCT scan was selected to include all upper and lower teeth and relevant anatomical structures. If patients had permanent metal restorations or implants, metal artefact reduction was applied. To reduce scatter at the occlusal plane, patients were instructed to bite on cotton rolls.

### Radiographic image processing—segmentation

**Fig. 1:**
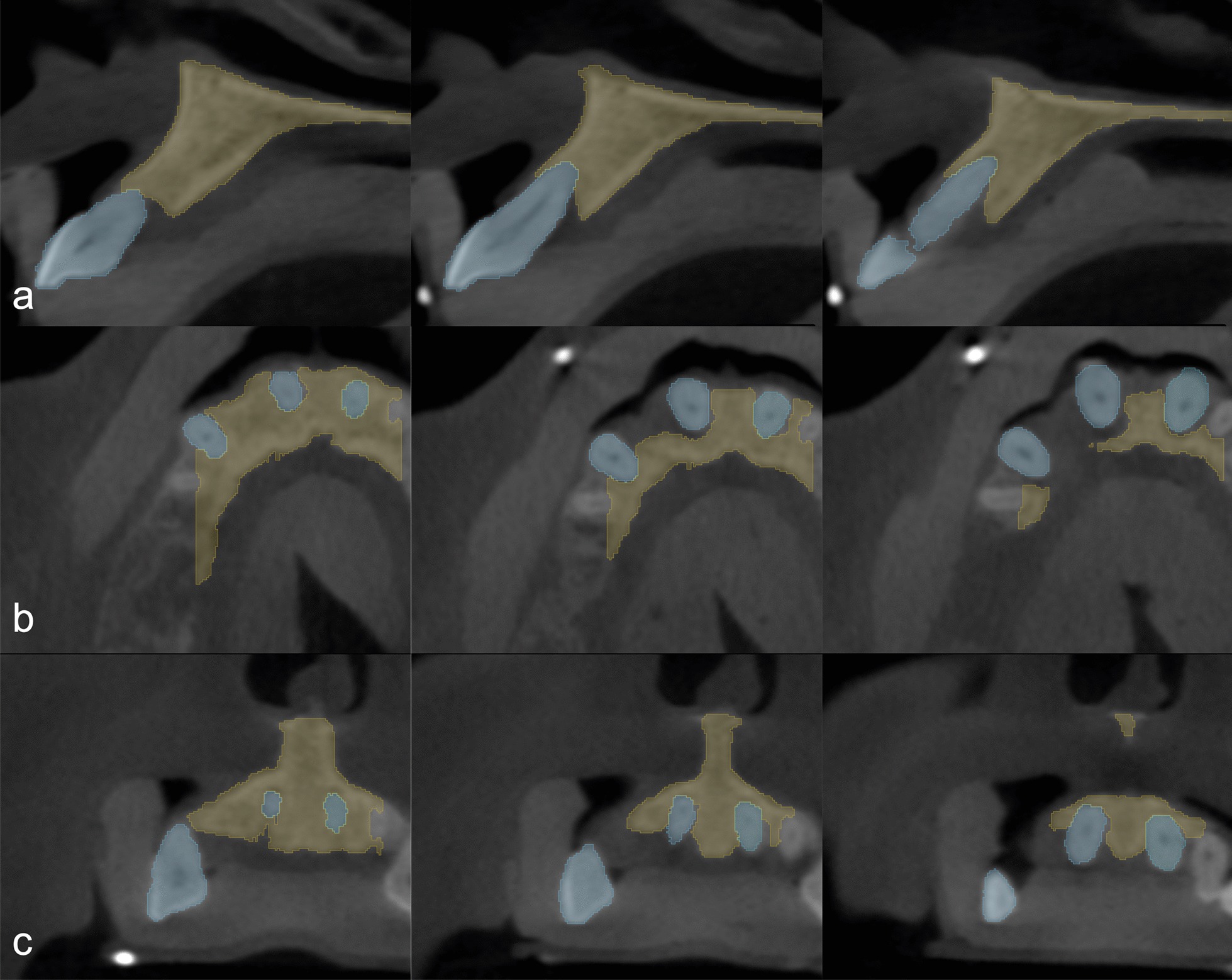
Regions of interest highlighted on slices of the CBCT dataset (brown: alveolar bone; blue: teeth). **a** Sagittal view. **b** Axial view. **c** Coronal view

**Fig. 2:**
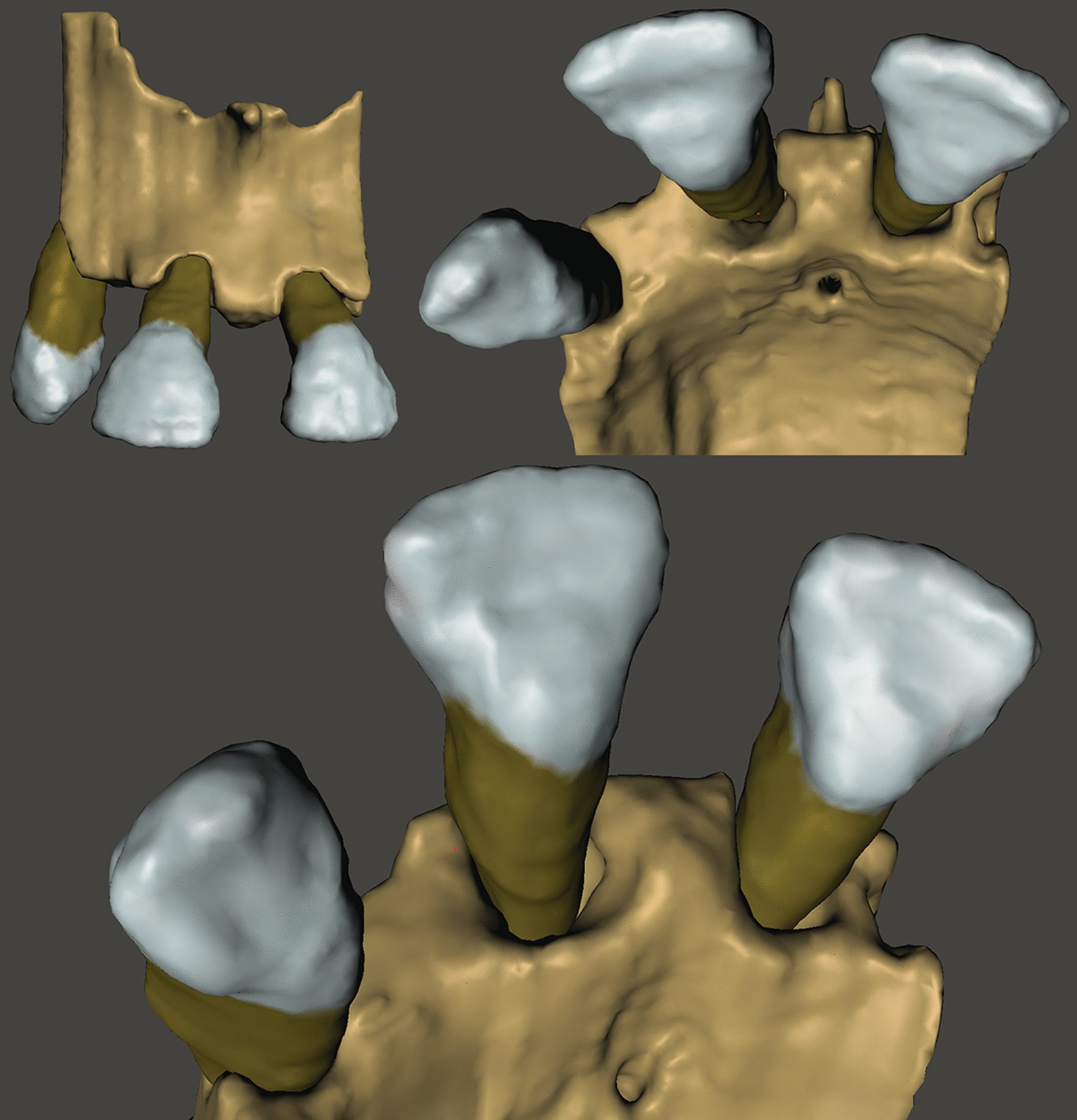
3D virtual model of periodontal defect, that aid the decision-making process prior to surgery. Horizonto-vertical defect at the palatal aspect of tooth 11, deep intrabony component at the mesial aspect, shallower towards the distal aspect, when eventually transitioning into the horizontal slope at the position of tooth 12

**Fig. 3:**
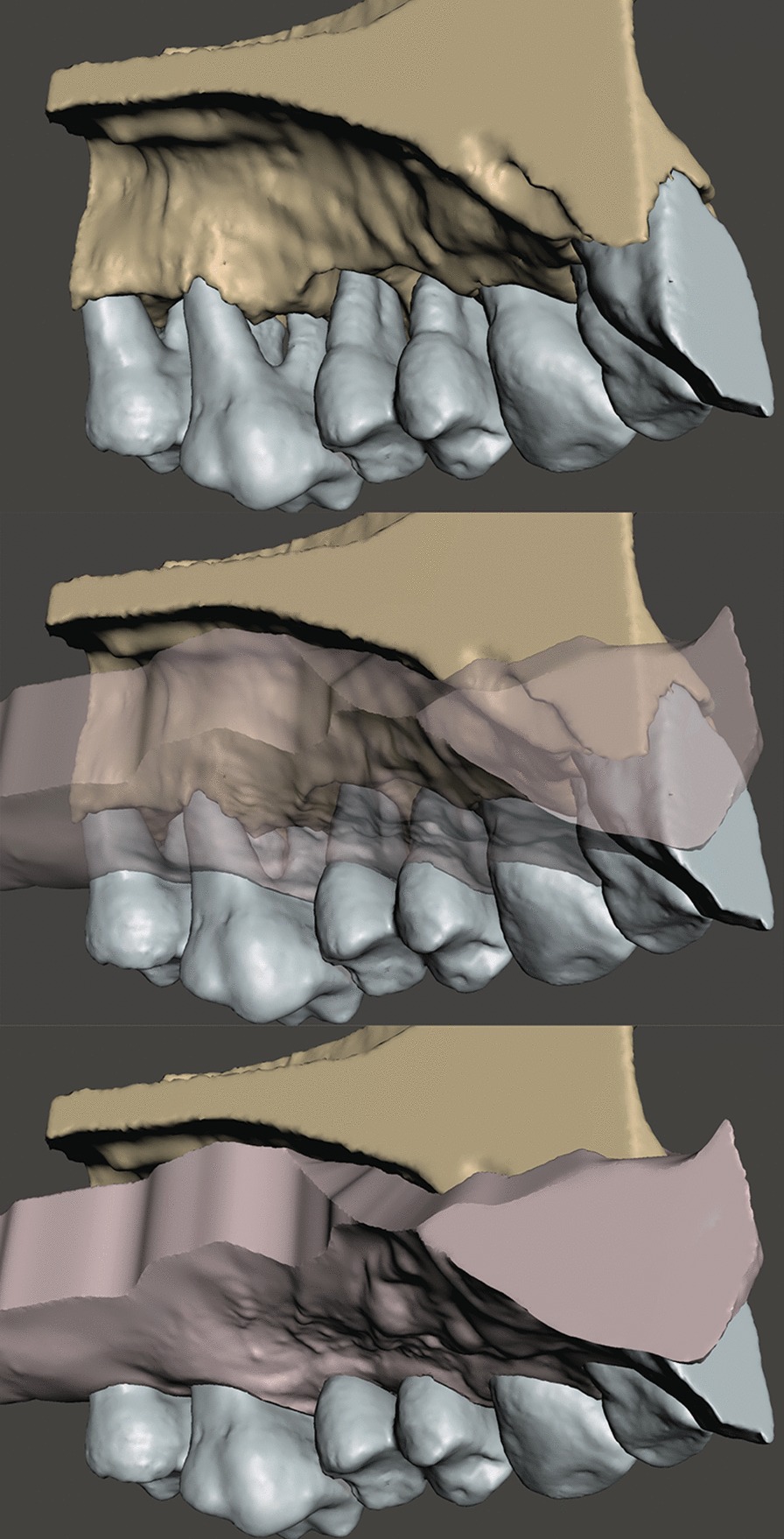
Soft tissue model derived from intraoral, hard tissue model reconstructed from CBCT. Circumdental crater around tooth 24 and 25, Class III furcation involvement of tooth 26, soft tissue model derived from an intraoral scan superimposed over the segmented model derived from CBCT dataset

DICOM (Digital Imaging and Communications in Medicine) datasets were imported into an open source medical image processing software (3D Slicer) [34] for image segmentation. The goal of segmentation was to generate 3D reconstructions of alveolar bone and teeth to allow easier analysis. Separate ROIs were created for the representation of alveolar bone and teeth. Various semi-automatic and manual segmentation tools were utilized. First, the *Level tracing* tool (used to outline a region where pixels have the same background value as the selected pixel) was applied at the alveolar bone on every 4th slice in the surgical field*.* Then, *Fill between slices* was applied utilizing morphological interpolation to calculate the missing ROIs [35]*.* Further refinement of individual ROIs was done with hand segmentation tools (*Paint brush, Erase, Scissors*). The same process was repeated for the teeth as well (Fig. 1). Three-dimensional polygon models were generated from the ROIs and were exported as stereolithographic *(.stl)* files. Further refinement and occasional mesh repairs were done with an open source computer-aided design (CAD) based mesh modelling software (Meshmixer^®^, Autodesk, San Rafael, California, USA) (Fig. 2). For more realistic digitalization of the clinical situation, a soft tissue model derived from an intraoral scan could be superimposed over the 3D model generated from the CBCT dataset (Fig. 3).

### Surgical procedure

**Fig. 4:**
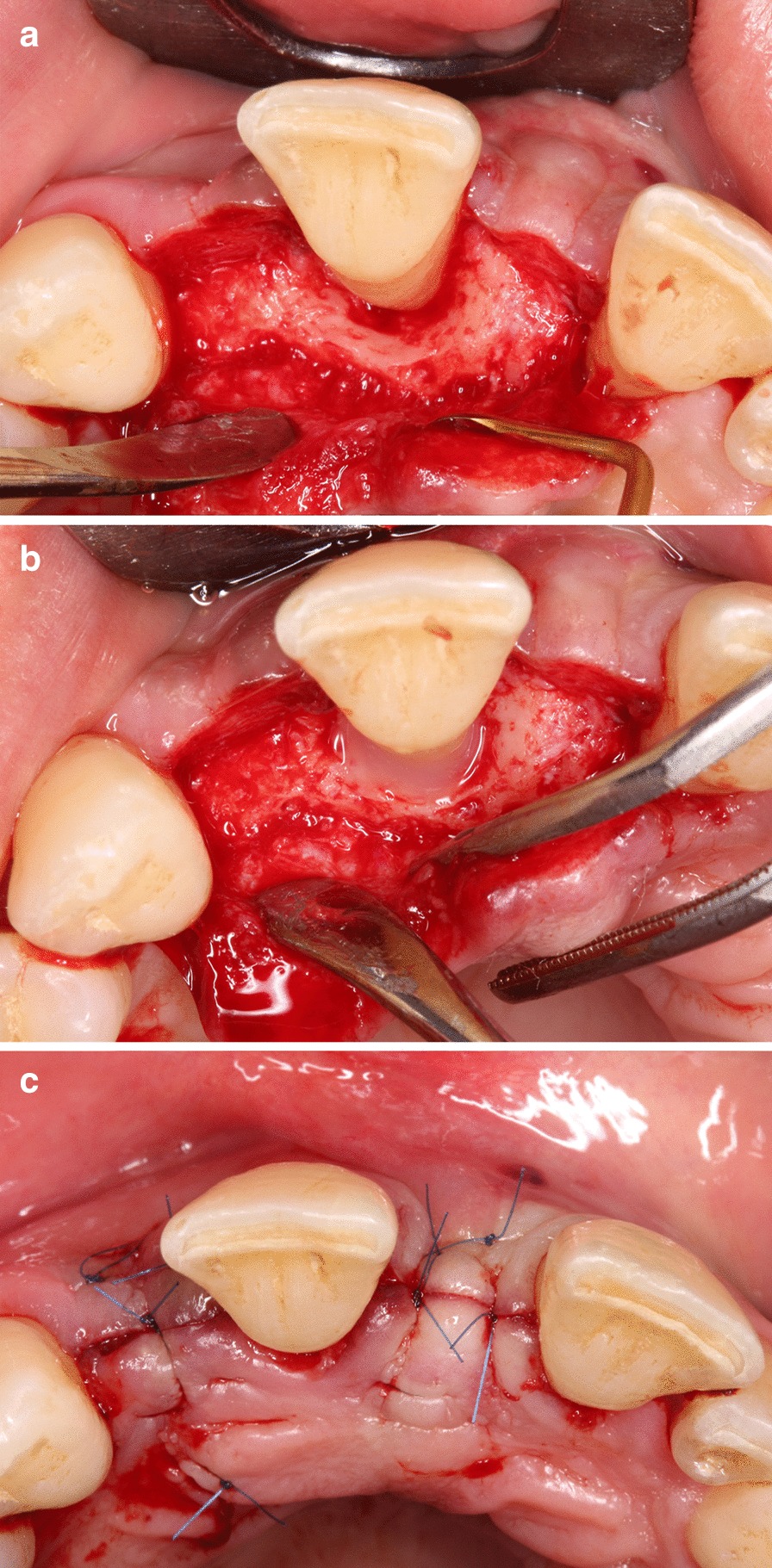
Regenerative periodontal surgery. **a** Palatal single flap approach. **b** Enamel matrix derivatives placed into the defect. **c** Double layer wound closure

Minimally invasive surgical procedures [2–4, 36] aim to improve blood clot and wound stability to enhance the regenerative potential and to reduce patient morbidity [37–39] by limited flap elevation. 3D visualization of the surgical area allowed for better understanding of the defect morphology, overcoming the limitations of reduced visibility during surgery. Periodontal defects were treated with either a single flap approach (SFA) [4] or a modified-minimally invasive surgical technique (M-MIST) [3]. The initial incision and flap elevation were made on the buccal or oral aspect, depending on the easiest access, predetermined on the virtual models. Following flap elevation, debridement of the defect was performed with hand and ultrasonic instruments. After instrumentation, root surfaces were conditioned with ethylenediaminetetraacetic acid (EDTA) (PrefGel^®^, Straumann, Basel, Switzerland) for 2 min. Enamel matrix derivatives (EMD) (Emdogain^®^, Straumann, Basel, Switzerland) were applied on a rinsed and dried root surface. Tension free wound closure was achieved by a double layer suturing technique with a non-resorbable 6–0 monofilament suturing material (Dafilon^®^, B Braun Melsungen, Germany) [4] (Fig. 4). Sutures were removed after 14 days. Following surgery, patients were instructed to avoid mechanical cleaning in the surgical area for 14 days. Participants were instructed to rinse twice per day for two weeks with 0.2% chlorhexidine (Curasept ADS 220^®^, Curaden International AG, Kriens, Switzerland). Following suture removal, patients were recalled at 1, 3, and 6 months.

### Outcome variables

**Fig. 5:**
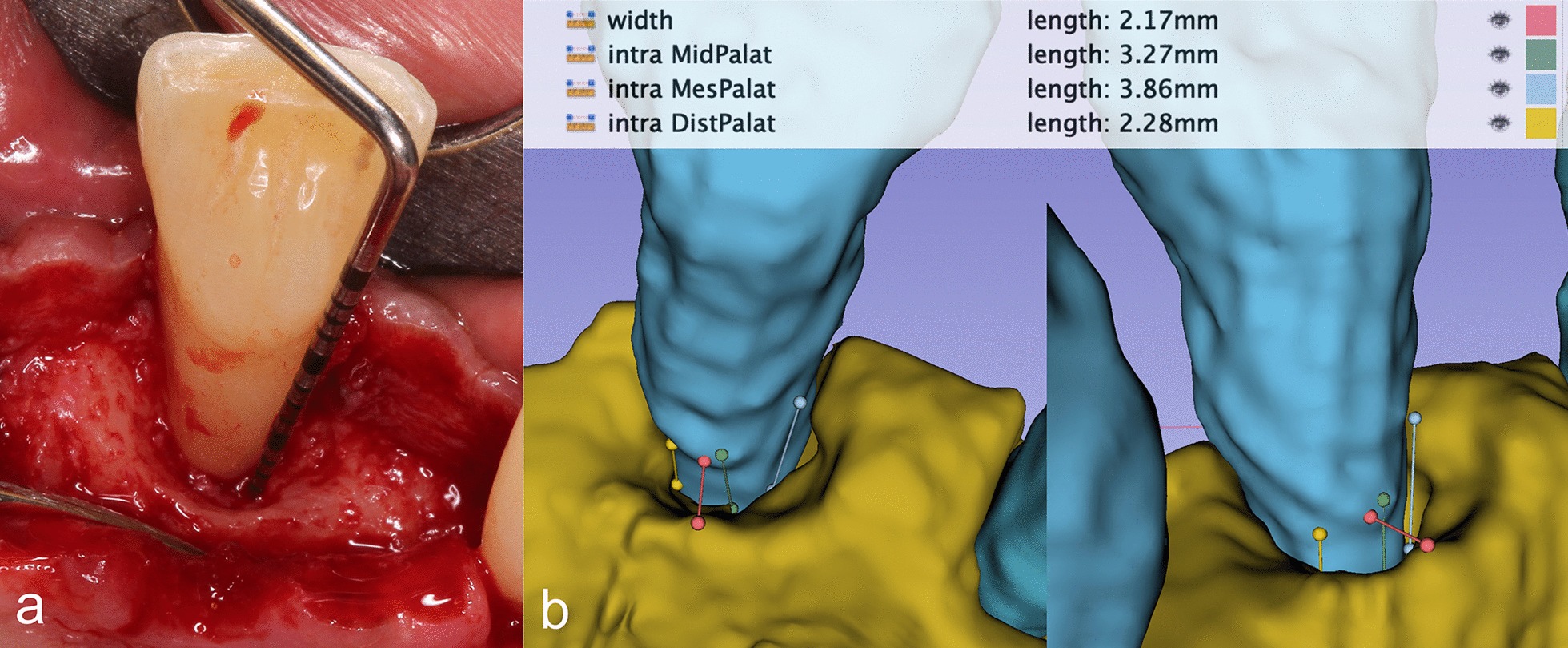
Comparing intrasurgical measurements with digital measurements. **a** Intrasurgical measurement. **b** Digital measurement

The primary outcome was to assess the accuracy of the 3D virtual models. Following flap elevation, intrasurgical measurements were performed at the affected tooth surfaces by one of the investigators to directly assess defect morphology. After surgical intervention, digital measurements were performed on the virtual models at the same aspects of the defects by a different examiner (Fig. 5). The vertical distance from the marginal bone crest to the base of the defect (intrabony component depth: INTRA) and the horizontal distance from the root surface to the most coronal point of the bone crest (root surface-bone crest distance: WIDTH) was registered intrasurgically [40].

**Fig. 6:**
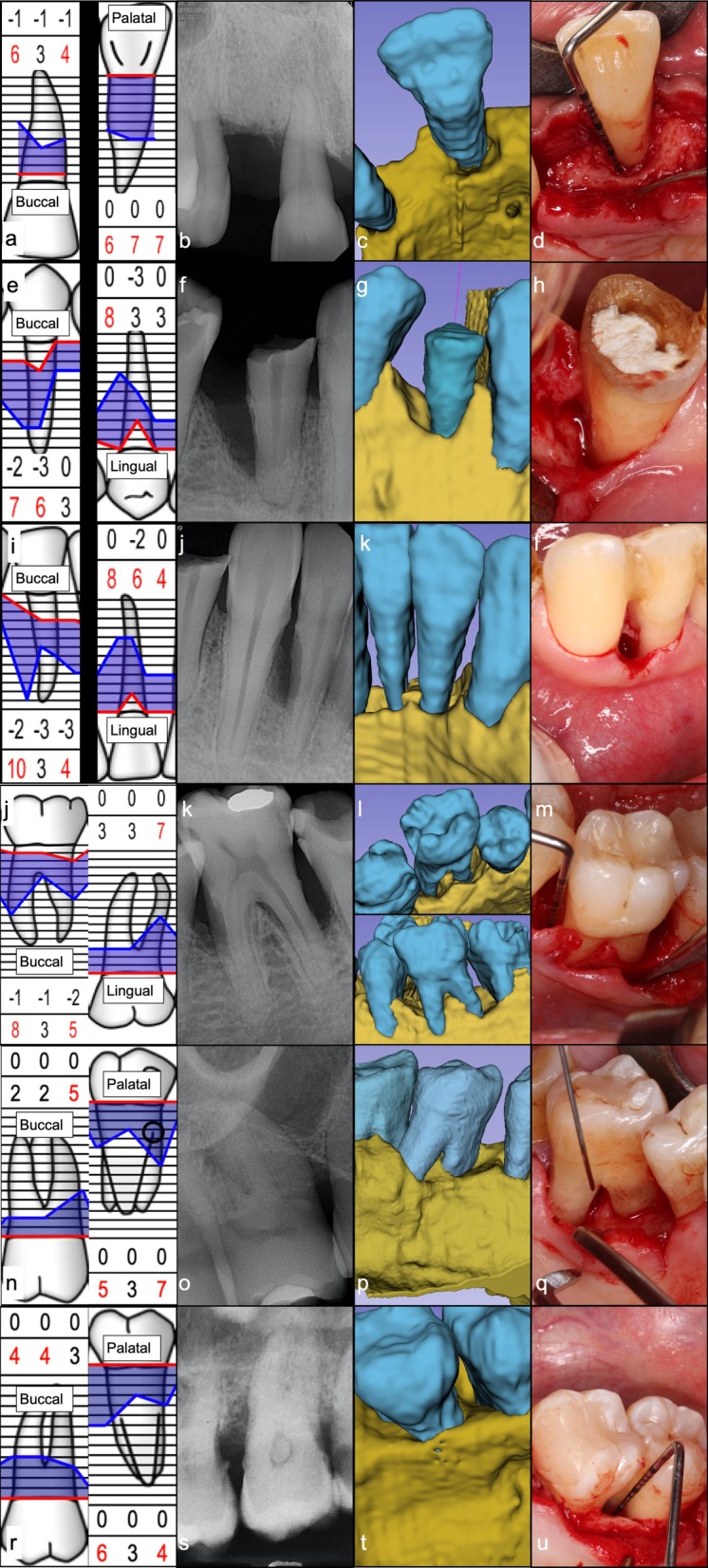
Intrabony periodontal defect morphology assessment with conventional diagnostic methods versus 3D virtual models. **a**–**d** Patient 1—Tooth 11. **e**–**h** Patient 2—Tooth 44. **i**–**l** Patient 2—Tooth 42. **j**–**m** Patient 2—Tooth 36. **n**–**q** Patient 3—Tooth 16. **r**–**u** Patient 4—Tooth 27

Additionally, it was investigated if defect characteristics (morphology, number of affected root surfaces, number of bony walls, furcation involvement) that determine the flap design and regenerative strategy [6] could be accurately predetermined by an experienced clinician with conventional diagnostic methods (intraoral radiographs and direct clinical measurements) (Fig. 6). In each case, if at least three of the four characteristics were determined correctly, the diagnosis was considered accurate (YES), and if two or less characteristics were determined correctly the diagnosis was considered inaccurate (NO).

Baseline clinical parameters were registered at baseline. FMPS and FMBS [41] were calculated as a percentage of the total tooth surfaces (six surfaces per tooth) to assess the oral hygiene and the level of active inflammation. PPD and GR were recorded at the surgical site with a UNC-15 periodontal probe. CAL was calculated for each measured surface.

### Data analysis

The data were expressed as mean value ± standard deviation. Primary focus of the statistical analysis was the differences between intraoperative and digital measurements. Intrasurgically and digitally measured values of the width and depth of intrabony components were compared and a one sample *t*-test was performed with a level of significance (*P*) set to 0,001. Statistical analysis was performed in SPSS Statistics^®^ (IBM, Armonk, New York, USA).

## Results

### Intrasurgical and digital measurements—validation of virtual model accuracy

Intrasurgically the depth and width of the intrabony component were measured (INTRA, WIDTH) at multiple aspects of the defects. Intrasurgically, the vertical distance from the marginal bone crest to the base of the defect averaged 4.22 ± 1.67 mm. On digital models the distance was 4.05 ± 1.51 mm. Horizontal distances between the marginal bone crest and the tooth surface measured at 3.17 ± 0.98 mm intrasurgically and 3.50 ± 1.02 mm on digital models. The difference between the width and depth of intrabony components were 0.31 ± 0.21 mm and 0.41 ± 0.44 mm respectively. The difference between intrasurgical measurements and digital measurements regarding the width and depth of the intrabony component of periodontal defects was statistically not significant (*P* > 0,001). Values and differences in intrasurgical and digital measurements are shown in Table 1.

**Table 1:** Comparison of intrasurgical and digital measurements

Defect	INTRA^a^ (mm)		WIDTH^b^ (mm)	
	Intrasurgical	Digital	Intrasurgical	Digital
1	3	2.28	2	2.17
	3	3.27		
	4	3.86		
2	8	7.41	4	4.36
3	3	3.23	2	2.25
	4	4.13		
4	5	4.72	4	3.75
5	3	2.80	4	4.15
6	5	4.79	3	4.30
	4.22 ± 1.64	4.05 ± 1.51	3.17 ± 0.98	3.50 ± 1.02

^a^Vertical distance from marginal bone crest to the base of the defect

^b^Horizontal distance from the root surface to the most coronal point of the bone crest

### Preoperative defect assessment

FMPS and FMBS values at baseline were 12.85 ± 3.90% and 9.73 ± 3.17% respectively. PPDs of 8.00 ± 1.26 mm and CALs of 9.67 ± 1.21 mm were recorded at baseline. Baseline values are shown in the Additional file 1.

Based on several defect characteristics (morphology, number of affected root surfaces, number of bony walls, furcation involvement), it was investigated if the defect morphology and extent could be predetermined precisely with conventional diagnostic methods versus on the 3D virtual models. Defect morphology was assessed accurately in only one case with IRs and direct clinical measurements. With conventional diagnostic methods (IRs + direct clinical measurements) 1- and 2-walled approximal intrabony components could be determined accurately. However, on the middle aspects of the teeth, defect characteristics were difficult to assess. Three furcation involved multi-rooted teeth were enrolled in the study. The grade of furcation involvement could not be determined precisely with conventional diagnostic methods. On the other hand, 3D models generated with the presented semi-automatic segmentation method depicted the defect characteristics correctly in all six cases. Data are shown in Table 2.

**Table 2:** Preoperative defect characteristics determined with conventional diagnostic methods and 3D virtual models

Defect characteristics		Patient 1	Patient 2			Patient 3	Patient 4
		Tooth 11	Tooth 44	Tooth 42	Tooth 36	Tooth 16	Tooth 27
Morphology	Convetional^a^	Horizontal	Vertical	Vertical	Vertical	Vertical	Horizontal
	3D^b^	Horizonto-vertical	Vertical	Vertical	Vertical	Vertical	Vertical
	Intraoperative	Horizonto-vertical	Vertical	Vertical	Vertical	Vertical	Vertical
No. of affected tooth surfaces	Conventional	3	1	1	2	1	1
	3D	3	1	2	3	2	3
	Intraoperative	3	1	2	3	2	3
No. of bony walls	Conventional	0	1	2	Mes^c^: 2; Dist^d^: 1	2	0
	3D	3	1	1	Mes: 1; Dist: 1	1	2
	Intraoperative	3	1	1	Mes: 1; Dist: 1	1	2
Furcation involvement	Conventional	–	–	–	Grade 0	Mesial grade I	Grade 0
	3D	–	–	–	Lingual grade II	Mesial grade II	Mesial grade II
	Intraoperative	–	–	–	Lingual grade II	Mesial grade II	Mesial grade II
Was it possible to determine defect characteristics with conventional methods?		NO	YES	NO	NO	NO	NO
Was it possible to determine defect characteristics with the aid of 3D virtual models?		YES	YES	YES	YES	YES	YES

^a^Direct clinical measurments + intraoral radiographs

^b^3D virtual models

^c^Mesial tooth surface

^d^Distal tooth surface

## Discussion

In this case series study, a novel semi-automatic segmentation method was utilized to generate realistic 3D virtual models for the planning of regenerative periodontal surgeries. Direct clinical measurements and IRs are the main diagnostic tools for periodontal surgical planning. However, it was concluded previously that in some cases, direct clinical measurements and IRs do not provide sufficient information on the morphology of intrabony periodontal defects, therefore surgical planning cannot be carried out properly [10, 11]. Application of CBCT scans has proven to be beneficial in the diagnostics of periodontal defects [15–22]; however, it should be used in cases where conventional methods are unable to provide an exact diagnosis [26]. In the present study, in five out of six cases conventional diagnostic methods did not provide sufficient information on defect characteristics. However, defect morphologies were successfully determined on the 3D virtual models in all cases, allowing for a more accurate treatment planning.

A limited number of articles described the application of 3D virtual models for periodontal treatment planning [27–29], in which CBCT scans were reconstructed with basic automatic thresholding algorithms without the application of a dedicated image reconstruction process. Various other automatic methods have been utilized for tooth and bone segmentation: (1) watershed algorithm [42]; (2) level set method [43]; and (3) convolution neural network [44]. The advantage of automatic segmentation is that it is less time consuming. However, different factors, such as metal artefacts and tissue conditions that differ from normal anatomy (i.e. periodontal bone loss), can compromise results [44]. The advantage of the presented semi-automatic segmentation method is the separate reconstruction of alveolar and dental structures to get a better understanding of 3D periodontal defect morphology.

A further drawback of the aforementioned automatic algorithms [42–44] is the limited access. They were programmed by individual groups for mainly personal use, and were never integrated into any commercially available software. In contrast, 3D Slicer is open source and free of charge, regularly receives updates, and has a wide variety of extensions developed for different applications that make it appealing for many users.

In the present article, dimensions of the intrabony component of defects measured digitally were compared to the intrasurgical measurements [40] and differences were calculated. Differences in the width and depth of the intrabony component averaged at 0.31 ± 0.21 mm and 0.41 ± 0.44 mm, respectively. Within the limitations of the present study, differences were found to be statistically not significant. However, further validation on larger sample sizes with standardized measurements has to be conducted.

One of the major concerns of using CBCT scans for periodontal diagnosis is the elevated radiation dose compared to conventional radiographic methods [23, 24]. The effective radiation dose of the applied large FOV I-CAT FLX^®^ scans was reported to be 83 µSv [45] which is only two times the dosage of a full-mouth intraoral periodontal status radiograph, reported to be about 40 µSv [46]. Minimally invasive flap designs were applied to reduce surgical trauma and postoperative patient morbidity [2–4]. Virtual models were also used for patient education to explain the clinical situation and the course of the surgery to the participants.

The presented semi-automatic segmentation method can be applied on any CBCT/ CT dataset. However, the quality of the 3D model is affected by the quality of the scans (larger voxel sizes, artefacts) [30]. A semi-automatic method was developed to achieve the highest possible quality of segmentation [47, 48] and even though the various semi-automatic tools and algorithms in the 3D Slicer database reduce the duration of the process, it is still very time-consuming.

In the current article, virtual models of only hard tissues were used for the surgical planning, therefore the clinical crowns of the teeth were not as detailed. To generate more realistic hybrid digital models, optical scans acquired with an intraoral scanner should be added on top of the hard tissue models for an even more precise planning process that will be expanded on in a future project.

To further expand on the concept of applying computer assisted technologies in periodontology, virtual models can be manufactured with rapid prototyping (3D printing) technologies. Realistic 3D printed models containing all relevant anatomical structures (teeth, bone, and gingiva) can be used for surgical simulation and during surgical intervention [49]. With 3D bioprinting technologies, defect-specific implants can be used as grafting materials [50]. Digital models, on the other hand, can also be uploaded into an augmented reality (AR) setup and be used with an AR headset [51] to further increase the visualization of the surgical field.

## Conclusions

It was found that the presented 3D virtual models acquired with the described semi-automatic segmentation method provide more accurate information on intrabony periodontal defect morphologies than conventional diagnostic methods. Values between intrasurgical and digital measurements were found to be not significant. It can be concluded that virtual models have depicted the actual defect morphology accurately. However, further validation on larger sample sizes with standardized measurements has to be conducted. In the current study, virtual models only contained hard tissues. However, soft tissue models acquired with an intraoral scanner can be added to generate more realistic virtual models that can be manufactured with 3D printing technologies and used for surgical simulation.

## Supplementary information

** Additional file 1** Baseline clinical measurements.

## Data Availability

Datasets used and analysed during the current study are available from the corresponding author on reasonable request.

## Abbreviations

PPD: Probing pocket depth
GR: Gingival recession
CAL: Clinical attachment loss
IR: Intraoral radiographs
2D: Two-dimensional
3D: Three-dimensional
CBCT: Cone-beam computed tomography
ROI: Region of interest
FMPS: Full mouth plaque score
FMBS: Full mouth bleeding score
FOV: Field-of-view
DICOM: Digital Imaging and Communications in Medicine
CAD: Computer aided design
SFA: Single flap approach
M-MIST: Modified minimally invasive surgical technique
EDTA: Ethylenediaminetetraacetic acid
EMD: Enamel matrix derivatives
CEJ: Cemento-enamel junction
BD: Base of defect
BC: Bone crest
mm: Millimeter
