# Supplementary material for: Digital three-dimensional visualization of intrabony periodontal defects for regenerative surgical treatment planning
Source: BMC Oral Health. 2020 Dec 1;20:351. doi: 10.1186/s12903-020-01342-w (PMC7709443; doi:10.1186/s12903-020-01342-w)
Supplement: Supplementary file 1 — Additional file 1 Baseline clinical measurements. [file 12903_2020_1342_MOESM1_ESM.docx]

| *Supplementary Table: Baseline clinical measurements* | | | | | | |
| --- | --- | --- | --- | --- | --- | --- |
| **Patient** | **Tooth** | **FMPS (%)** | **FMBS (%)** | **PPD (mm)** | **GR (mm)** | **CAL (mm)** |
| 1 | 11 | 11.3 | 5.4 | 7 | 2 | 9 |
| 2 | 44 | 8.7 | 10.5 | 7 | 2 | 9 |
|  | 42 |  |  | 10 | 2 | 12 |
|  | 36 |  |  | 8 | 1 | 9 |
| 3 | 16 | 17.9 | 13 | 7 | 3 | 10 |
| 4 | 27 | 13.5 | 10 | 9 | 0 | 9 |
|  |  | ***12.85 ± 3.90*** | ***9.73 ± 3.17*** | ***8.00 ± 1.26*** | ***1.67 ± 1.03*** | ***9.67 ± 1.21*** |
